# Arthroscopic ankle arthrodesis for end-stage ankle osteoarthritis

**DOI:** 10.1530/EOR-2023-0100

**Published:** 2025-05-05

**Authors:** E Carlos Rodríguez-Merchán, William J Ribbans, José M Olmo-Jiménez, Alberto D Delgado-Martínez

**Affiliations:** ^1^ Department of Orthopaedic Surgery, La Paz University Hospital, Madrid, Spain; ^2^ Osteoarticular Surgery Research, Hospital La Paz Institute for Health Research – IdiPAZ (La Paz University Hospital – Autonomous University of Madrid), Madrid, Spain; ^3^ Faculty of Health, Education and Society, University of Northampton, Northampton, UK; ^4^ The County Clinic, Northampton, UK; ^5^ Department of Orthopaedic Surgery, Hospital Universitario de Jaén, Jaén, Spain; ^6^ Department of Surgery, University of Jaén, Jaén, Spain

**Keywords:** ankle, osteoarthritis, arthroscopic arthrodesis, results

## Abstract

- Arthroscopic ankle arthrodesis (AAA) has been performed for 40 years for end-stage ankle osteoarthritis. Along with open ankle arthrodesis (OAA) and total ankle replacement (TAR), it forms one arm of the triumvirate of commonly performed procedures for this condition.
- The aim of this article is to review the state of the art for AAA and compare outcomes with OAA and TAR.
- This narrative review of the literature traces the development of this technique through case series and systematic reviews.
- Traditional OAA techniques carry a nonunion rate of 11%, necessitating revision surgery in most cases. As individual and communal experience of AAA has grown, the range of pathology and deformity successfully corrected by this technique has developed.
- There is evidence that AAA offers greater and more rapid union rates, with reduced hospital stay and better long-term outcomes. However, the technique requires mature surgical skills and still carries a significant complication rate.
- No single procedure is suitable for all patients. AAA can be seen as the new gold standard for patients with isolated ankle osteoarthritis and no/minimal deformity, either within the talocrural joint or hindfoot or patients with systemic and/or local comorbidities that would benefit from minimal disturbance to the soft-tissue envelope.
- However, in older patients, the presence of concomitant hindfoot osteoarthritis or significant deformity, TAR and OAA remain valuable procedures in the foot and ankle surgeon’s armamentarium.

## Introduction

Ankle osteoarthritis etiology differs from hip and knee pathology, with primary ankle osteoarthritis being less common (7% of cases) (1). Posttraumatic (70%) and inflammatory arthritis (12%) are the most common causes (1). It tends to present in younger patients compared to more proximal lower limb articulations (2).

Once conservative options have been exhausted, surgical options including arthrodesis, total ankle replacement (TAR) and, less commonly, osteotomy need to be considered. Open ankle arthrodesis (OAA) has historically achieved reliable results. The first description of arthroscopic ankle arthrodesis (AAA) was reported by Shneider in 1983 (3). Subsequently, many authors have reported that AAA has advantages over open arthrodesis (4, 5, 6, 7, 8, 9, 10, 11, 12, 13, 14, 15, 16, 17, 18, 19, 20, 21).

The purpose of this article is to review the state of art for AAA and compare outcomes with OAA and TARs. In this article, we have analyzed AAA for end-stage ankle osteoarthritis. A PubMed (MEDLINE) search of studies related to AAA for end-stage ankle osteoarthritis was performed. The key words used were ‘arthroscopic ankle arthrodesis’. The main inclusion criteria were articles focused on AAA (indications/contraindications, surgical technique, outcomes and complications) and comparative studies of AAA with OAA and TAR. Studies not focused on the aforementioned topics were excluded. The search included papers up until 2024. A total of 341 papers were identified, of which 69 fulfilled the necessary criteria for inclusion.

## Indications and contraindications for AAA

Surgical indications for AAA demonstrated no commonality in the reviewed publications. Ferkel & Hewitt recommended a minimum of 6 months conservative treatment before embarking upon an AAA (13). Authors have varied in their indications for AAA, including primary osteoarthritis (4, 12, 13, 14, 15, 17), posttraumatic osteoarthritis (5, 11, 12, 18), inflammatory arthritis (5, 12, 18), avascular necrosis (AVN) of the talus (11, 12), and neuromuscular deformities (18). AAA is most commonly performed in posttraumatic, relatively young patients (21). AAA’s smaller surgical wounds are preferable in the presence of comorbidities such as vascular disease, diabetes mellitus and local issues such as previous infection, excessive scarring or fragile soft tissues. Contraindications include active infection (13), and severe AVN of the talus and/or large bone defects (11, 22).

Surgeons need to be competent ankle arthroscopists. A learning curve is attached to the procedure. Some authors advocate the use of AAA in cases of no/minimal deformity only (4, 5, 8, 11, 13, 14), while others extend the indication to more severe deformities (15). Previously, a coronal tibiotalar angle of 15° had been advised as the upper limit for correction by AAA (3, 7, 23), but technique familiarity has allowed progressively larger correction of deformities during AAA (12, 15, 24, 25). The presence of concomitant osteoarthritis in the hindfoot or midfoot may favor a TAR to preserve movement. The indications and contraindications for AAA in the literature are summarized in Table 1.

**Table 1:** Indications and contraindications for AAA.

Indications	Contraindications
• End-stage osteoarthritis of the ankle (primary osteoarthritis, osteoarthritis with minimal or no deformity of the ankle, osteoarthritis with marked deformity of the ankle)	• Active infection • Severe avascular necrosis of the talus • Large bone defects
• Posttraumatic osteoarthritis (sequelae of tibial pylon fracture or due to chronic instability after lateral ligament injury)	
• Rheumatoid arthritis	
• Osteochondritis dissecans of the talus	
• Avascular necrosis of the talus (≤30% of the talus)	
• Neuromuscular deformity of the ankle	

AAA, arthroscopic ankle arthrodesis.

## Surgical technique of AAA

**Figure 1:**
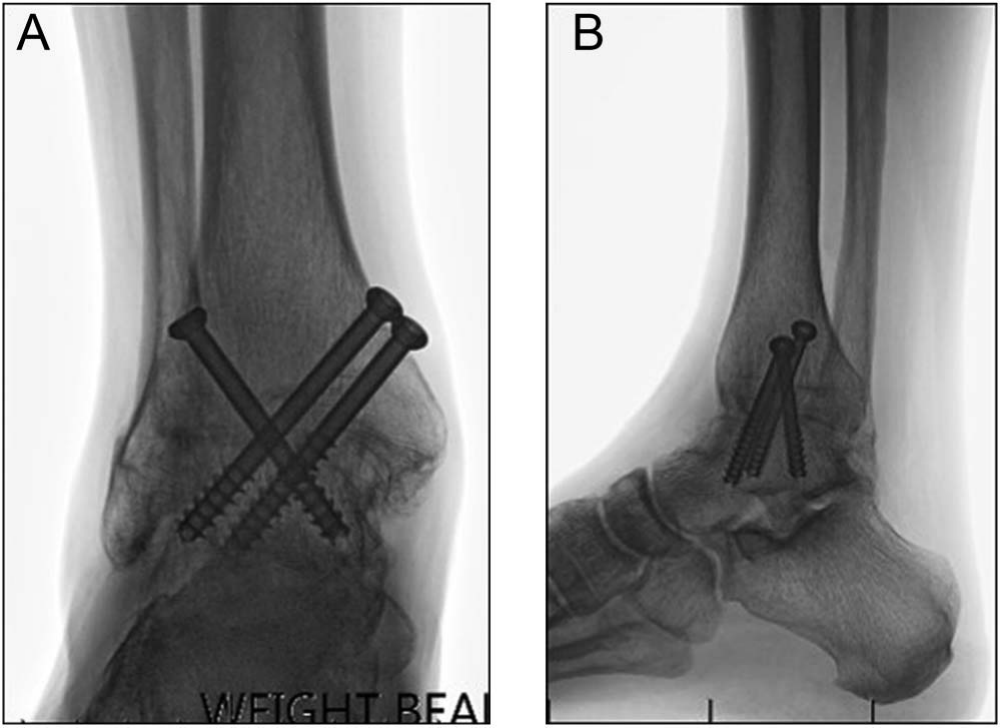
Radiographs taken 12 months after arthroscopic ankle arthrodesis (AAA) in a 55-year-old man: (A) anteroposterior view; (B) lateral view.

AAA requires thorough preparation and excision of degenerate cartilage, followed by percutaneous screw fixation across the joint (19). The number of arthroscopic portals utilized is variable. Standard anteromedial and anterolateral portals are frequently supplemented by additional portals medially, laterally and posteriorly to improve access. The size, number and placement of fixation screws vary from two to five (Fig. 1) (4, 5, 7, 14, 16, 19, 21). Some authors augment with autograft, allograft or bone graft substitutes to stimulate healing. Additional procedures including Achilles or gastrocnemius lengthening or foot osteotomies may be required to correct deformity and improve postsurgical foot positioning. For both OAA and AAA, optimal postoperative limb biomechanics are achieved by fusing the ankle in neutral dorsiflexion with a posteriorly translated talus and 5–10° of hindfoot valgus and external rotation (26).

To optimize implant placement, Park *et al.* (2020) (27) described a novel technique using external fixators and cannulated screws to construct a 3-dimensional navigation drill guide to predict screw trajectory prior to insertion, to avert metalwork impingement. The technique can assist surgeons in implementing percutaneous screws for AAA (27). In 2022, Guo *et al.* achieved good results using a pin-based distractor to open and close the tibiotalar interspace, correct the talar tilt and maintain a good mechanical axis for fusion (28). In 2023, Yokoo *et al.* described that larger sagittal inter-screw distance to tibial width ratios resulted in diminished post-AAA delayed union or nonunion. Surgeons should be aware that the anterior and posterior screw widths should be around 60% or more of the anteroposterior width of the tibia (29).

**Figure 2:**
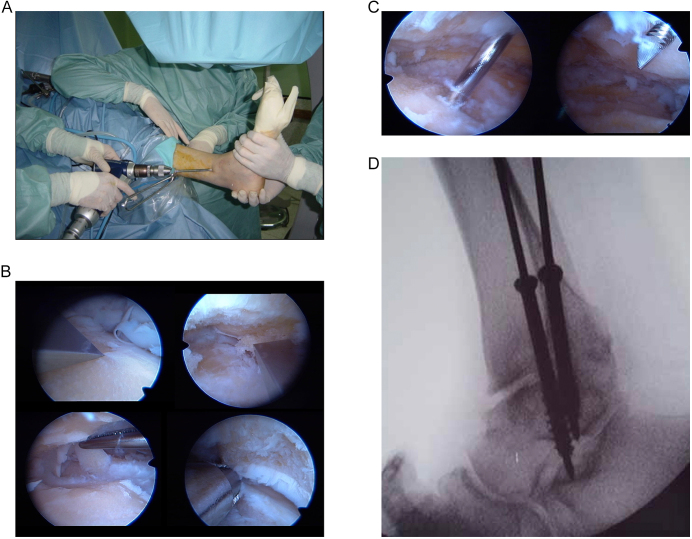
(A, B, C, D) Images of a case of arthroscopic ankle arthrodesis: (A) arthroscopic set up for the procedure; (B) preparation and excision of degenerate cartilage; (C) introduction of guide pins for percutaneous cannulated screws; (D) cannulated screws in final position.

Other authors have performed arthroscopic tibiotalocalcaneal arthrodesis procedures (TTCA). In 2020, Baumbach *et al.* reported that although their results with arthroscopic TTCA did not prove compared to open TTCA, there seemed to be a strong trend towards considerably lower complication rates following arthroscopic TTCA (30). In 2022, Guerra Alvarez *et al.* found that posterior arthroscopic TTCA offered very good results, with a high rate of bony union, few adverse events and reduced non-weight-bearing time. They recommended using this technique for individuals without major deformities, especially in those at high risk of adverse events related to the surgical wound (31). Table 2 summarizes the fundamental steps of the AAA surgical technique (21). Fig. 2 shows the main surgical steps of AAA.

**Table 2:** Surgical technique of arthroscopic ankle arthrodesis (AAA).

**Instruments required**
4.0 mm arthroscope (30°) or 2.9 mm arthroscope (30°)
Arthroscopic resector and bur
Osteotomes and curettes
Short and long partially and fully threaded screws (6.7 mm or 6.5 mm cannulated screws)
Ankle distractor strap
**Portals**
Standard AM and AL portals are utilized and tagged on the skin at the level of the ankle joint line. The AM portal must be placed just medial to the anterior tibial tendon
During the surgical procedure, supplementary portals such as the medial and lateral gutter portals will be needed. In some cases, accessory PM (between tibialis posterior and flexor digitorum longus) and PL (posterior to the peroneals) might be needed to prepare the posterior talus
The ankle is insufflated with 20 cc normal saline via the medial approach. The AM and AL portals are carried out utilizing a nick and spread technique
If an anterior osteophyte of great size exists, fluoroscopy may have to be utilized to identify the joint. Then, a bur, curette or osteotome can be utilized to debride the osteophyte. The soft tissues must be elevated off the osteophyte in order not to damage the anterior neurovascular structures. Besides, removal of the anterior osteophyte can permit for the dorsiflexion of the talus and better neutral positioning of the talus in the sagittal plane
**Preparation of the joint**
The cartilage of the talus and tibia should be removed with a soft tissue resector or bur, followed by curettes
If the surgeon chooses to prepare the gutters, medial and lateral gutter portals can be utilized to clear the medial and lateral side of the talus, as well as the medial side of the distal fibula and the lateral side of the medial malleolus, from cartilage
The articular surfaces must be prepared with a bur to get bleeding, punctate subchondral bone
Besides, small golf ball-like punctures have to be made with the high-speed bur to create zones of spot welds
A chisel can be utilized to create AP rails to further break the subchondral bone
All debris must be removed with the help of the soft tissue resector
**Addressing the deformity**
If a large defect exists, an iliac crest bone graft must be harvested. A structural iliac crest allograft can also be utilized
A tricortical wedge must be formed, with the adequate height to correct the existing deformity
Soft tissue release must be carried out if required
The tricortical wedge is then inserted either over a short AL or AM incision by extending the portals
Distances of less than 1.5 cm can be bridged with fully threaded screws to strut out the deformity and shield potential collapse
**Positioning the screws**
The alignment of the ankle is then corrected to 5° of valgus, neutral dorsiflexion and 5° to 10° external rotation. This position must be fixed with Kirschner wires and confirmed with intraoperative fluoroscopy in AP and lateral planes
The screw placement is then carried out, beginning with the compression screw, which counteracts the deformity. In general, a minimum of two screws must be utilized
Definitive fluoroscopy views in AP, mortise, lateral of the ankle, dorsoplantar and oblique of the foot must be made to verify the correct position of the arthrodesis and the good position and length of the screws

AM, anteromedial; AL, anterolateral; PM, posteromedial; PL, posterolateral; AP, anteroposterior; AAA, arthroscopic ankle arthrodesis.

## Outcomes of AAA

Review of the outcomes of AAA is derived from both case series and systematic reviews. By placing significant case series in historical order, the evolution of the indications, techniques and outcomes is better appreciated.

### Case series

Table 3 shows the most relevant information from the case series published on AAA results (4, 5, 6, 7, 8, 9, 10, 11, 12, 13, 14, 15, 17, 18, 19, 20).

**Table 3:** Outcomes of AAA: case series.

Study	Year	Methods	Results	Conclusions
Dent *et al.* (5)	1992	Eight procedures (eight patients). No patient had severe ankle deformity	Clinical fusion was achieved in all cases	Radiological evidence of bone fusion was accomplished in four cases
Ogilvie-Harris *et al.* (4)	1993	19 procedures (19 patients)	15 had radiographic evidence of union within 3 months and further two by 6 months	AAA produced good results in osteoarthritic patients with no/minimal deformity
Crosby *et al.* (6)	1996	42 procedures (41 patients). A bi-framed distraction technique augmented with DBM slurry was used	At mean FU of 27 months, 85% were satisfied	DBM did not appear to improve fusion rates compared to previously non-augmented AAAs
Glick *et al.* (7)	1996	34 procedures (34 patients). Mean FU: 8 years	The fusion rate was 97%; 86% had good or excellent results. There were no wound infections or neurological injuries	Fusion time was significantly shorter than OAA techniques, which accelerated the recovery period
Jerosch *et al.* (8)	1996	26 procedures (26 patients). Patients' age ranging from 31–69. FU: 6–75 months	85% were solidly fusion at the time of final follow-up. 50% achieved fusion within 3 months	AAA was recommended for degenerative joint disease without rotational or varus/valgus malalignment, severe bone defects or neuropathic disease
Fisher *et al.* (9)	1997	15 procedures (15 patients)	13 AAAs fused. 11 patients underwent outpatient surgery	The procedure was considered safe and effective with less patient morbidity than OAA
Cameron *et al.* (10)	2000	15 procedures (15 patients). FU: 1–3 years	All fused at an average of 11.5 weeks	The procedure had a significant complication rate
Zvijac *et al.* (11)	2002	21 procedures (21 patients). Mean age: 52.7 years. Mean FU: 34 months	Fusion occurred in 20/21 patients with a mean time to clinical and radiographic union of 8.9 weeks	Compared to OAA, AAA advantages were a high fusion rate, shorter time to fusion and lower cost
Winson *et al.* (12)	2005	105 procedures (104 patients). Preoperative talocrural deformity ranged from 22° valgus to 28° varus	Mean age: 57 years. Mean FU: 65 months	Mean union time was 12 weeks. Clinical outcomes: 79% good-excellent, 10% fair, 11% poor
Ferkel & Hewitt (13)	2005	35 procedures (35 patients). Mean FU: 72 months	Overall fusion rate was 97%. Mean time to fusion was 12 weeks	This study demonstrated a high fusion rate with minimal complications for AAA.
Collman *et al.* (14)	2006	39 procedures (in 39 patients with minimal/no ankle deformity)	87% achieved union at a mean time of 47 days	The addition of DBM or PRP did not improve union rate
Gougoulias *et al.* (15)	2007	78 procedures (in 74 patients with a range of deformities)	48 had minor deformity (group A) and 30 had a varus or valgus deformity >15° (maximum 45°) (group B)	Outcomes were rated as very good in 79% of group A and 80% of group B, fair in 19% of group A and 17% of group B and poor in one ankle in each group
Jain *et al.* (17)	2014	52 procedures (52 patients). Mean age: 59.4 years. Mean FU: 32 months	92% achieved radiographic union at a mean time of 12 weeks	Patients with neuromuscular problems may require more rigid fixation to achieve consistent union rates
Kolodziej *et al.* (18)	2017	25 procedures (25 patients). Mean FU: 26 months	Fusion occurred in 92% with a mean of 12 weeks	NA
Vaishya *et al.* (19)	2017	28 procedures (28 patients)	Minimum FU of 1 year (mean 1.7 years). Mean age: 43.7 years; 64% were male. Mean hospital stay was 2 days	All cases fused at a mean time of 14 weeks. 71% were graded excellent, 14% good, 11% fair and one patient with a poor clinical outcome
Jones *et al.* (20)	2018	101 procedures (101 patients). Mean age: 61.1 years. Mean FU: 86 months	95% of patients achieved radiographic fusion. The AOS scoring system showed 75% good/excellent results. 96% of patients returned to work after AAA	AAA appeared effective in treating degenerative ankle disease, even in the presence of moderate coronal tibiotalar deformity

DBM, demineralized bone matrix; FU, follow-up; OAA, open arthroscopic arthrodesis; PRP, platelet-rich plasma; NA, not available; AOS, ankle osteoarthritis scale; AAA, arthroscopic ankle arthrodesis.

### Systematic reviews

In a systematic review with level IV evidence, Abicht & Roukis investigated the incidence of AAA nonunion (16). Study inclusion criteria included isolated ankle arthrodesis, more than 20 ankles, minimum 12-month follow-up, 2-portal anterior arthroscopic approach, fixation with two or three large-diameter cannulated cancellous screws and a published nonunion rate without restriction of cause. Seven articles met the inclusion criteria. A total of 244 ankles were included. Sixty-one percent were male, with a mean age of 49 years. Mean follow-up was 24 months. An 8.6% nonunion rate was reported, with 67% of these being painful and requiring revision surgery.

Yasui *et al*. undertook a systematic review in 2016 (32). The authors reviewed papers on OAA and AAA. Having applied their exclusion criteria, 26 OAA and 16 AAA papers were reviewed. Overall AAA fusion rates at 94% were better than OAA at 89%.

## Complications of arthroscopic ankle arthrodesis (AAA)

AAA is not without complications. All series reported nonunion rates. This outcome is regarded as the ‘benchmark’ for various arthrodesis techniques. However, the rate of other complications is less clear. Most published series are retrospective and differ in the assiduousness of reporting. By reviewing fifteen series (4, 5, 6, 7, 8, 9, 10, 11, 12, 13, 14, 15, 17, 18, 19), a pooled cohort of 561 patients could be examined. Reported complication rates must be regarded as the lower limit for reasons stated above. Foot and ankle arthroscopy in general has a significant complication rate ranging from 2.7 to 17% (33, 34, 35, 36, 37, 38).

An AAA nonunion rate of 6.6% was reported in our 561 patients. Yasui *et al*. concluded similarly (6%; range 0–30%) (32). Malunion was described in 0.9% of cases. Deep infection rates were 0.5% and superficial infection rates 0.9%. In the only series using external fixation (6), there was a 10% pin site infection rate. Fractures were reported in 0.5%. Hardware issues were variously reported as ‘metalwork issues’, fixation revision and metalwork removal. Such problems were reported in 9.1%. Similarly, problems with adjacent hindfoot joints (predominantly but not exclusively the subtalar articulation) were described variously as pain and/or requiring fusion. Cumulatively, this involved 2% of patients. Venous thromboembolic (VTE) pathology was reported in 0.5%. Huntley *et al.* (2018) reported an identical VTE rate after arthroscopy (39), and Griffiths *et al*. reported an incidence of 0.4–1.4% in elective foot and ankle surgery (40). Neurological issues appeared underreported, with superficial peroneal nerve (SPN) injury and complex regional pain syndrome (CRPS) each only reported in 0.1% of patient. Ferkel *et al.* self-reported an overall 4.4% rate of neurological complications following ankle arthroscopy for any indication, with SPN injury alone at 2.5% (41). Rewhorn *et al.* reported a 4.4% incidence of CRPS after elective foot and ankle surgery. Eleven (2.0%) further unspecified complications were stated (42).

Overall, 148 problems were recorded (26.4%), with some patients sustaining more than one complication. The complications of AAA in the literature are summarized in Table 4.

**Table 4:** Potential complications of AAA.

Complications
• Nonunion – some requiring revision procedures
• Fractures
• Pin site infections
• Superficial infection
• Deep infection – requiring antibiotics and/or surgical intervention
• Symptomatic hardware – some requiring removal and/or revision of metalwork
• Symptomatic subtalar joint osteoarthritis – some requiring later subtalar fusion
• Malunion
• Correct foot alignment not achieved
• Deep vein thrombosis/pulmonary embolism
• Neurological damage, e.g. SPN and CRPS

AAA, arthroscopic ankle arthrodesis; SPN, superficial peroneal nerve; CRPS, complex regional pain syndrome.

## Comparison of AAA with OAA and TAR

Several comparative studies of AAA with OAA, both systematic reviews and case series, have been published. Table 5 summarizes the most salient information from these comparative studies published in the form of systematic reviews (43, 44, 45). Table 6 summarizes relevant information from comparative case series studies (46, 47, 48, 49, 50). Table 7 shows the results of AAA comparing coronal deformity of less than 15° versus coronal deformity equal to or greater than 15° (case series) (51, 52).

**Table 5:** Comparative systematic reviews and meta-analyses: AAA versus OAA.

Study	Year	Findings
Mok *et al.* (43)	2020	AAA was associated with a greater fusion percentage, shorter estimated blood loss, smaller tourniquet time and shorter LOS than OAA
Bai *et al.* (44)	2021	AAA had more advantages than OAA in ameliorating the fusion percentage and diminishing adverse events
Xing *et al.* (45)	2023	AAA was better than OAA alone in the management of ankle osteoarthritis based on the percentage of adverse events, intraoperative tourniquet time, LOS, nonunion percentage and rate to fusion

AAA, arthroscopic ankle arthrodesis; LOS, length of the hospital stay; OAA, open ankle arthrodesis.

**Table 6:** AAA versus OAA: comparative studies (case series).

Study	Year	Patients and methods	Results	Conclusions
Woo *et al.* (46)	2020	28 AAAs (28 patients) versus 56 OAAs (56 patients) for age, sex and BMI. VAS scores, AOFAS Ankle-Hindfoot Scale scores and SF-36 evaluated prior to surgery, and at 6 and 24 months following surgery	The AAA cohort showed significant less pain in the perioperative period. Individuals who experienced AAA also reported a higher SF-36 score on physical functioning at 6 months and higher AOFAS Ankle-Hindfoot Scale score at 2 years. There were no postoperative adverse events in the AAA cohort but 11 in the OAA cohort, including 9 which needed follow-up operations	The AAA cohort exhibited better clinical results compared to the OAA cohort at the 2 years follow-up. The pros of AAA included substantially less perioperative pain, greater AOFAS Ankle-Hindfoot scores at 2 years, smaller LOS, fewer postoperative adverse events and follow-up operations
Morelli *et al.* (47)	2021	Cohort A (OAA; 11 procedures in 11 patients) versus cohort B (AAA, 12 procedures in 12 patients); the two cohorts were homogeneous with regard to age and BMI. The AOFAS, FAS and VAS for pain intensity were assessed preoperatively, at 6 months and at final follow-up of 7.6 years in cohort A and 7.3 years in cohort B	Individuals in the AAA cohort exhibited better outcomes at 6-month follow-up compared to the OAA cohort at the AOFAS and the FAS scores. Pain alleviation was accomplished in both cohorts at 6-month follow-up. Both cohorts exhibited improved clinical results from baseline to final follow-up. LOS was smaller in cohort B than in cohort A. More adverse events were found in the open cohort than in the arthroscopic cohort	AAA and OAA are valid and safe alternatives for the treatment of ankle osteoarthritis on the basis of clinical results at 7 years follow-up. AAA showed faster improvement at 6-month follow-up in comparison with OAA
Martinelli *et al.* (48)	2022	23 OAAs (23 patients) versus 21 AAAs (21 patients)	Union rate was greater (90.5 versus 65.2%) and adverse event rate was lower (14.3 versus 47.8%) in the AAA cohort. Individuals who experienced AAA reported better pain relief, with higher improvements in VAS for pain scores. No substantial difference in length of operative time, time to fusion, AOFAS, and FFI scores improvements between the two cohorts was found	AAA led to higher union percentages, fewer adverse events, and lower percentages of reoperation in individuals at high risk of adverse events
Shah *et al.* (49)	2022	46 OAAs (46 patients) versus 41 AAAs (41 patients)	The nonunion rate was 11% in the OAA and 12% in the AAA cohort. In the AAA cohort, a remote history of infection and the utilization of headed screws had notably greater risk of nonunion (not statistically significant). In the OAA cohort, utilization of bone graft trended toward lower risk of non-union	Similar percentages of non-union between OAA and AAA in high-risk individuals were found. For individuals with a remote history of infection, OAA might be preferable
Abuhantash *et al.* (50)	2022	223 AAAs (223 patients) versus 128 OAAs (128 patients). The two cohorts were similar preoperatively with respect to demographics, but COFAS type-4 arthritis was relatively more frequent in the OAA cohort and type-1 osteoarthritis was relatively more frequent in the AAA cohort. AOS score and AAS were better in the AAA cohort	The differences in postoperative outcome scores between the cohorts were not substantial. The risk of revision due to mal-union or non-union was comparable in both cohorts (6% in the AAA cohort, compared with 4% in the OAA cohort). Deep infection and wound adverse events did not happen in the AAA cohort but happened in 4% of the individuals in the OAA cohort	There were no differences in PROMs between the two surgical procedures. AAA had a similar revision rate but lower infection rate than OAA

AAA, arthroscopic ankle arthrodesis; OAA, open ankle arthrodesis; BMI, body mass index; VAS, visual analog scale; AOFAS, American Orthopedic Foot and Ankle Society; SF-36, Short Form Health Survey; LOS, length of hospital stay; N, number of procedures; FAS, Freiburg Ankle score; FFI, Foot Function Index; ASA, American Society of Anesthesiology; COFAS, Canadian Orthopedic Foot and Ankle Society; PROMs, patient reported outcome measures.

**Table 7:** AAA: coronal deformity of less than 15° versus coronal deformity ≥15° (case series).

Study	Year	Patients and methods	Results	Conclusion
Yang *et al.* (51)	2020	41 AAAs (41 patients) were performed with three cannulated screws. Mean FU: 51.4 months. All individuals were 60 years of age or older (men age: 70.6 years). Demographic data and radiographic and functional results were compared between individuals with coronal deformity of less than 15° (cohort I, *n* = 26) and those with a deformity equal to or greater than 15° (cohort II, *n* = 15). LOE: III, RCS	Near-normal tibiotalar alignment was accomplished postoperatively in both cohorts. Union was accomplished in 39 (95.1%) individuals with two cases in cohort I experiencing nonunion. Union rate, mean AOFAS Ankle-Hindfoot Scale, and VAS pain scores were not significantly different between the two cohorts at final FU	AAA was a dependable surgical technique for end-stage ankle osteoarthritis in individuals 60 years of age or older leading to a high union percentage, encouraging radiographic and functional results, and a low percentage of adverse events, even in cases with severe preoperative deformity. Arthroscopic intra-articular malleolar osteotomy was a useful surgical procedure for correcting severe coronal deformity in this series
Issac *et al.* (52)	2022	122 AAAs (122 patients). These were divided into two cohorts; cohort A (*n* = 99) with deformity less than 15° and cohort B (*n* = 23) with deformity ≥15°. The mean FU in cohort A and B was 74.9 and 89.2 months respectively. The average deformity in cohort A was 4.9° for AAA and 5.8° for OAA. In cohort B it was 18.9° (maximum 28° varus) for AAA and 22.1° (maximum 41° valgus) for OAA. LOE: level III, RCS	The overall union rate was 95% in cohort A (AAA: 94%; OAA: 100%) and 87% in cohort B (AAA: 100%; OAA: 67%). Mean time to union was 13.2 weeks in cohort A (AAA: 13.3 weeks; OAA: 12.8 weeks) compared to 12.4 weeks for cohort B (AAA: 12.9 weeks; OAA: 11.8 weeks). Irrespective of the intensity of deformity and type of surgical procedure, smokers had a 10 times higher probability of nonunion. In cohort A, none of the PROMs exhibited significant difference between AAA and OAA. In cohort B, EQ-VAS score reached statistical significance in favor of AAA whereas other PROMs exhibited no difference	AAA was reproducible in accomplishing union in end stage ankle osteoarthritis and good PROMs can be expected even in ankles with intense deformities. Regardless of the type of surgical procedure and intensity of deformity, smoking was a significant risk factor for non-union

AAA, arthroscopic, ankle arthrodesis; AOFAS, American Orthopedic Foot and Ankle Society; FU, follow-up; LOE, level of evidence; RCS, retrospective comparative series; VAS, visual analog scale; PROMs, patient-related outcome measures; EQ-VAS, EuroQol visual analog scale.

A difficulty in comparing outcomes of the three procedures is matching primary pathology, comorbidities and deformity degree pre-surgery. In addition, many case series are retrospective and lacking technique comparisons.

The systematic review by Yasui *et al*. reported a 5% lower union rate for OAA at 89% (32). Improved comparisons are derived from series containing both techniques performed by the same surgeon. Such pooled results show a closer union rate of 89% (OAA) to 91% (AAA) (22, 32, 53, 54, 55). Reports indicate AAA results in shorter hospital stays (5, 22, 54) and union times (7, 11, 22, 53, 55, 56). In addition, AAA has reported better clinical outcomes (55, 57, 58).

TARs are undertaken less often than total hip replacements (THRs) and total knee replacements (TKRs). In the 2019 UK National Joint Registry report, 1,004 TARs were undertaken in 2018 compared to 106,116 THRs and 109,540 TKRs (59). This reflects both the relative frequency of end-stage osteoarthritis in these three joints and the presence of acceptable alternatives for the ankle, i.e., AAA and OAA.

Studies including outcomes between TAR and some form of arthrodesis are infrequent (60, 61, 62, 63, 64, 65, 66). The outcomes of the UK-based TARVA study (total ankle replacement versus arthrodesis), when reported, will provide the first level 1 study (67). Reviews of comparative trials do not usually describe the form of arthrodesis or contain mixed methods. Only Veljkovic *et al*. directly compared distinct AAA and OAA groups to TAR. In this study, TAR and AAA procedures had similar outcomes, at a mean of 43 months follow-up, in patients selected without intra- or extra-talocrural deformity or concomitant hindfoot osteoarthritis. However, TAR patients were subjected to a greater number of later, further additional procedures (66). A recent TAR systematic review reported an overall complication rate of 23.7% (range: 2.4–52%) with more than one-third rated as high-grade. TAR re-operation levels were 17.4% (68).

Yasui *et al*. reported that OAA resulted in a greater incidence of postsurgical hindfoot arthritis than AAA (with a relative odds ratio of 2.17) (32). It is not clear if this represents ‘*de novo*’ or exacerbated pre-existing arthritis. Ling *et al*. concluded that there was no clear evidence that ankle fusion caused or exacerbated hindfoot osteoarthritis (69).

In the UK, 29,000 patients annually present to tertiary care for advice about ankle osteoarthritis. 10% will be offered surgical intervention (67). Clearly not all patients suit one surgical approach for treating end-stage ankle osteoarthritis. The elderly patient with concomitant hindfoot osteoarthritis would benefit from the retention of some ankle movement brought by a TAR. Gross ankle deformity would suit an OAA approach whereas high-demand patients with no/moderate deformity or those with significant systemic or local comorbidities might be advised to undergo an AAA.

## Conclusion

AAA has been undertaken for 40 years. As individual and collective experience has developed, increasingly more challenging cases (in terms of deformity and comorbidities) have been undertaken. Experience is required for optimal results and the technique has a learning curve. Union rates are at 93–94%. However, despite the problems of underreporting, overall complications (minor and major) are estimated to be at 26%. Fusion rates appear marginally better than OAA with reduced hospital stays, fusion times and recovery. There is no evidence that TAR surgery is superior to AAA. AAA can be undertaken in patients with comorbidities that would preclude TAR such as vascular disease, poorly controlled diabetes and compromised local soft tissues. AAA has proven itself to be an effective procedure in proficient hands for a range of pathologies and capable of correcting at least moderate coronal deformities. In this cohort, it should be regarded as the new gold standard for end-stage ankle osteoarthritis. However, AAA, OAA and TAR should all be part of the armamentarium of experienced foot and ankle surgeons in considering options for this group of patients.

## ICMJE Statement of Interest

The authors declare that there is no conflict of interest that could be perceived as prejudicing the impartiality of the research reported.

## Funding Statement

This research did not receive any specific grant from any funding agency in the public, commercial or not-for-profit sector.
